# The OFD1 protein is a novel player in selective autophagy: another tile to the cilia/autophagy puzzle

**DOI:** 10.15698/cst2021.03.244

**Published:** 2021-02-17

**Authors:** Manuela Morleo, Brunella Franco

**Affiliations:** 1 Telethon Institute of Genetics and Medicine (TIGEM), Via Campi Flegrei, 34, 80078, Pozzuoli, Naples, Italy.; 2 Medical Genetics, Department of Translational Medical Sciences, University of Naples Federico II, Via S Pansini 5, 80131, Naples, Italy.

**Keywords:** OFD1, Selective autophagy, Autophagy receptor, Primary cilium, ULK1 complex, Oral-Facial-Digital type I syndrome, Renal cystic disease

## Abstract

The autophagy-lysosomal pathway is one of the main degradative routes which cells use to balance sources of energy. A number of proteins orchestrate the formation of autophagosomes, membranous organelles instrumental in autophagy. Selective autophagy, involving the recognition and removal of specific targets, is mediated by autophagy receptors, which recognize cargos and the autophagosomal membrane protein LC3 for lysosomal degradation. Recently, bidirectional crosstalk has emerged between autophagy and primary cilia, microtubule-based sensory organelles extending from cells and anchored by the basal body, derived from the mother centriole of the centrosome. The molecular mechanisms underlying the direct role of autophagic proteins in cilia biology and, conversely, the impact of this organelle in autophagy remains elusive. Recently, we uncovered the molecular mechanism by which the centrosomal/basal body protein OFD1 controls the LC3-mediated autophagic cascade. In particular, we demonstrated that OFD1 acts as a selective autophagy receptor by regulating the turnover of unc-51-like kinase (ULK1) complex, which plays a crucial role in the initiation steps of autophagosome biogenesis. Moreover, we showed that patients with a genetic condition caused by mutations in *OFD1* and associated with cilia dysfunction, display excessive autophagy and we demonstrated that autophagy inhibition significantly ameliorates the renal cystic phenotype in a conditional mouse model recapitulating the features of the disease (Morleo et al. 2020, EMBO J, doi: 10.15252/embj.2020105120). We speculate that abnormal autophagy may underlie some of the clinical manifestations observed in the disorders ascribed to cilia dysfunction.

Autophagy is an evolutionary conserved and highly regulated cellular degradation pathway which entails the engulfment of cellular components into double-membrane vesicles, called autophagosomes, which then fuse with lysosomes to degrade their content and recycle nutrients back into the cytoplasm. Autophagy is active at basal levels in most cell types where it plays a housekeeping role in maintaining the integrity of intracellular organelles and proteins. It is also, however, strongly induced by nutrient depletion to promote survival until nutrients become available again. Bulk autophagy is non-selective, while selective autophagy involves the recognition and removal of specific targets through autophagy receptors. Autophagy receptors link their cargo to autophagosomes through LC3B-interacting regions (LIR) that mediate binding to autophagosomal membrane proteins LC3 and/or GABARAP, thus enabling cargo sequestration and degradation together with their own receptors.

Autophagy, both basal and selective, plays a variety of roles in physiological and pathological conditions. Both insufficient and excessive levels of autophagy can have detrimental effects. Indeed, this process has a protective effect in neurodegeneration and lysosomal storage disorders; conversely, excessive activation of autophagy can contribute to cancer.

Recent studies have shown that primary cilia and ciliary signaling pathways control autophagy and that, conversely, autophagy is one of the main players in the regulation of ciliogenesis. Primary cilia are microtubule-based organelles protruding from the cell surface of mammalian cells and display sensory functions. In post-mitotic cells, the centrosome, composed of two centrioles and of centriolar satellites, moves to the apical cell surface where the mother centriole docks with the plasma membrane and subtends axonemal microtubules of primary cilia. Centrioles participating in ciliogenesis and located under the ciliary axoneme are called basal bodies. Ciliary dysfunction has been implicated in disorders called “ciliopathies” which present overlapping phenotypes, such as primary ciliary dyskinesia, retinal degeneration, cystic disease, skeletal defects, situs inversus, obesity, mental retardation, and central nervous system (CNS) malformations.

Pampliega and colleagues described that both mature autophagosome markers and autophagy proteins implicated in the initial steps of autophagosome formation localize to cilia or periciliary regions. The authors proposed that the ciliary membrane could act as a nucleation site for pre-autophagosome formation. Moreover, their report and others showed that autophagy activation requires the presence of a functional primary cilium. On the other hand, it has been shown that autophagy influences ciliogenesis in a cellular context dependent manner. In particular, selective autophagic degradation of the satellite pool of OFD1 that physiologically acts as a cilia inhibitor, promotes ciliogenesis. OFD1 is a centrosomal/basal body protein whose centriolar fraction is instead required for primary cilia formation. However, different laboratories have reported contrasting results, suggesting that the role played by this degradative pathway in ciliogenesis might be cell context dependent.

The impact of this sensing organelle in autophagy and conversely, the molecular mechanisms underlying the direct role of core autophagic proteins in cilia biology, remains elusive.

In our recent study we revealed the molecular mechanisms underlying the direct functional role of the centrosomal/basal body protein OFD1, in LC3-mediated autophagy control, and the far-reaching implications for ciliopathies. We demonstrated that OFD1 is a novel selective autophagy receptor which controls the early phases of the autophagic cascade (**Figure 1**). We found that OFD1 controls the turnover of the unc-51-like kinase (ULK1) complex, which plays a crucial role in the initiation steps of autophagosome biogenesis and comprises the protein kinase ULK1 and the regulatory components ATG13, FIP200 and ATG101. In particular we demonstrated that OFD1 interacts at centrosomes with LC3B and GABARAP-L1 through a classical LIR motif and with ATG13 thus promoting its LIR-mediated autophagic degradation. We demonstrated through pharmacological and genetic approaches that ATG13 is an autophagy target, and that ATG13 molecules are less abundant in the lumen of swollen lysosomes in human kidney knock out (KO)-*OFD1* cells compared to controls. In addition, we confirmed that OFD1 is degraded through autophagy and demonstrated that the autophagy-mediated degradation of OFD1 is dependent on its LIR. Altogether, our data demonstrate that OFD1 is a novel autophagy receptor, which selectively promotes the autophagic degradation of ATG13 via direct interaction with the Atg8/LC3/GABARAP family of proteins. These observations raise the question of the physiological role of OFD1 as an autophagy receptor. We tested the ULK1 kinase activity in *OFD1* depleted cells and observed that the ULK1 complex enhanced protein stability was associated with increased ULK1 kinase activity. Consistent with this model, analysis of autophagy confirmed that loss of OFD1 increased the number of autophagosome vesicles. We also showed, by monitoring lysosome-mediated autophagosome clearance and the levels of the autophagic substrate p62 that OFD1 depletion enhances overall autophagic flux. Furthermore, we verified that the increased autophagy observed in KO-*OFD1* cells is independent of the mammalian target of rapamycin kinase pathway, the main negative regulator of autophagy. Conversely, OFD1 overexpression resulted in reduced autophagosomes biogenesis, which was dependent on OFD1-LC3/GABARAP binding through LIR, confirming its role in selective autophagy-mediated inhibition of autophagosome biogenesis.

**Figure 1:**
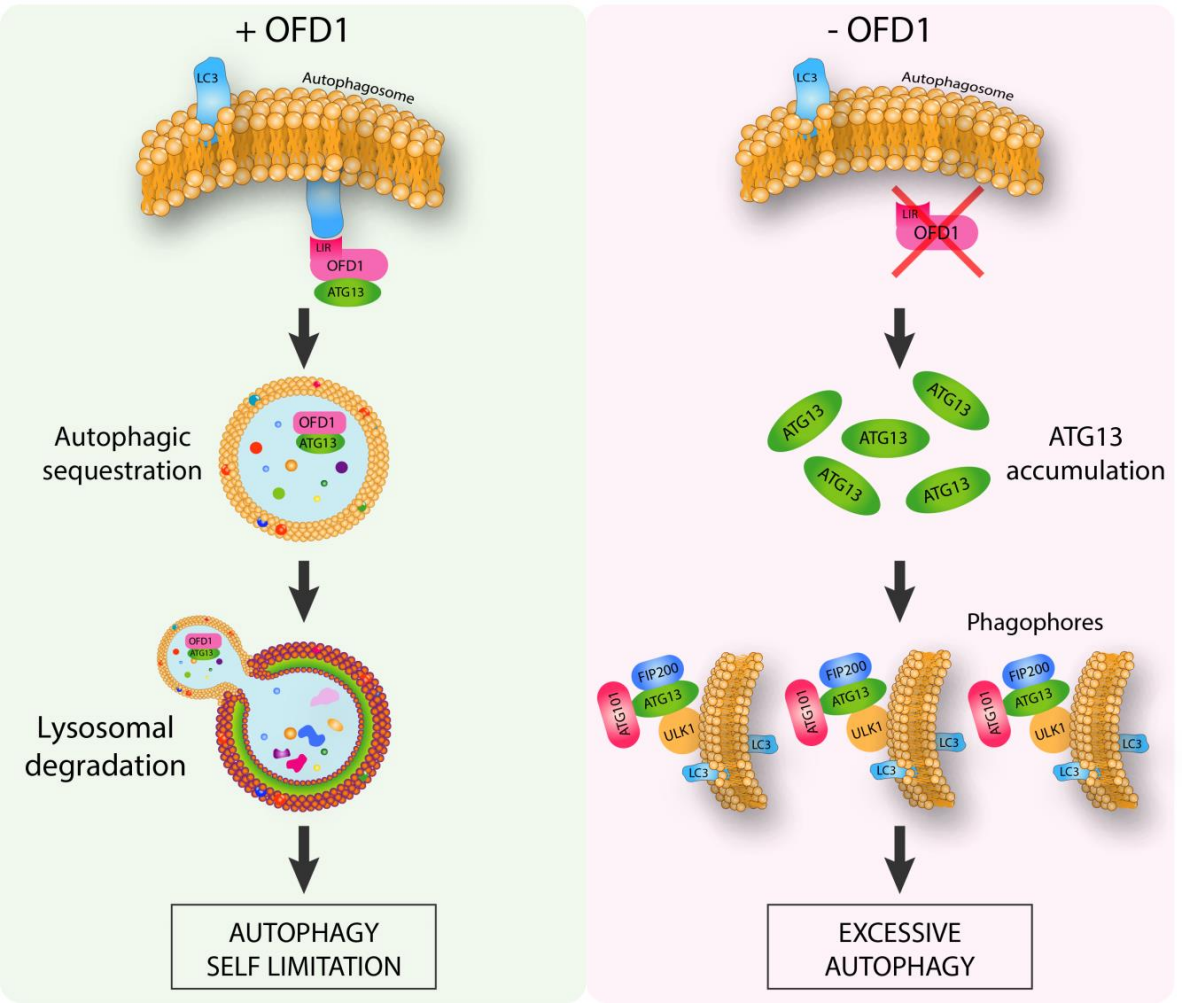
FIGURE 1: The centrosomal protein OFD1 controls autophagy. OFD1 promotes the selective autophagy-mediated degradation of ATG13, a component of ULK1 autophagy initiation complex. Loss of OFD1 determines ULK1 complex enhanced protein stability associated with excessive autophagy (Adapted from Morleo M et al. (2020), EMBO J, doi: 10.15252/embj.2020105120).

In addition, we also demonstrated that OFD1 controls autophagy independently from cilia: KO-*OFD1* cells that cannot form primary cilia reproduced the same autophagic phenotype in conditions promoting ciliogenesis (i.e. mitotic quiescent and confluent cells), and in those not allowing cilia formation (i.e. cycling and subconfluent cells). This observation allows us to speculate that the OFD1 protein could be regarded as a novel noncanonical autophagic player controlling autophagy, independently from its role in cilia biology. On the other hand, we cannot exclude that the autophagy receptor OFD1 could selectively recognize other cargoes besides ATG13. We speculate that OFD1 could act as a novel autophagic receptor for ciliary proteins exerting either a positive or negative effect on ciliogenesis and mediate their selective degradation to promote or inhibit ciliogenesis in a cell context dependent fashion. Recent evidence reporting that different cilia components such as IFT88, ARL13, centrin 1, pericentrin, PCM1 and CEP131 are degraded through autophagy, support this hypothesis. OFD1 could be one of the players coordinating the deep and intricate bidirectional crosstalk between autophagy and cilia that could explain the need for the cell to compartmentalize autophagic and ciliary proteins to the same cellular area.

However, the mechanisms underlying OFD1-mediated selective autophagic degradation of the effectors of ciliogenesis are yet to be determined and could involve more autophagy receptors. To validate this hypothesis, studies exploring the interaction of OFD1 with LC3 and other OFD1-interacting proteins are required to identify and characterize the potential substrate/s of the autophagy pathway that use/s OFD1 as receptor. Experimental evidence which shows that different cilia components, such as centriolar satellites proteins PCM1 and CEP131, are autophagic substrates and physically interact with LC3 and GABARAP support this hypothesis.

Mutations in the *OFD1* gene are responsible for Oral-Facial-Digital type I syndrome (OFD type I, OMIM 311200), a dominant male lethal X-linked ciliopathy characterized by the presence of renal cystic disease in 50% of cases. We showed that patients with OFD type I syndrome display excessive autophagy and that genetic inhibition of autophagy in a mouse model recapitulating the features of the disease significantly ameliorates the renal phenotype, implicating excessive autophagy in the pathogenesis of renal cysts. Nevertheless, we cannot exclude that abnormal autophagy could have a role not only in cystic kidney but also in other clinical manifestations, such as skeletal abnormalities and CNS involvement, and that ciliopathies should be considered autophagic diseases. This consideration implies that other genes encoding centrosomal/basal body and ciliary proteins could have a role in autophagy and that their mutations could be associated with abnormalities of autophagic flux. Further studies on the characterization of autophagy in other ciliopathy models will be necessary to prove if abnormal autophagy could underlie some of the clinical manifestations observed in disorders ascribed to cilia dysfunction. It is tempting to speculate that abnormal function of centrosomes/centriolar satellites/basal body/ciliary proteins could be associated with autophagy-related disorders such as cancer, neurodegenerative, muscle, and liver diseases.

Further understanding of the molecular mechanisms underlying the functional interactions between OFD1 and autophagy will be of utmost importance not only from a basic science point of view but also for the possible therapeutic implications for ciliopathies and other conditions.

